# The complete mitochondrial genome of the terebellid polychaete *Neoamphitrite affinis* (Polychaeta; Terebellidae)

**DOI:** 10.1080/23802359.2022.2136981

**Published:** 2022-10-30

**Authors:** Sang-Eun Nam, Somyeong Lee, Yeonhui Lee, Jae-Sung Rhee

**Affiliations:** a Department of Marine Science, College of Natural Sciences, Incheon National University, Incheon, South Korea; b Research Institute of Basic Sciences, Incheon National University, Incheon, South Korea; c Yellow Sea Research Institute, Incheon, South Korea

**Keywords:** Complete mitogenome, Terebellida, Terebellidae, *Neoamphitrite affinis*, polychaete

## Abstract

Here, we sequenced and annotated the complete mitochondrial genome for the terebellid polychaete, *Neoamphitrite affinis* (Malmgren 1866). The complete mitogenome of *N. affinis* is 15,823 bp, with 33.4% A, 18.2% C, 11.5% G, and 37.0% T. The circular *N. affinis* mitochondrial genome comprises 13 protein-coding genes (PCGs), 23 transfer RNA (tRNA) genes including 2 methionine tRNA genes, 2 ribosomal RNA (rRNA) genes, and a non-coding region. Phylogenomic analysis based on 26 in-group taxa belonging to the two main clades, Sedentaria and Errantia, is congruent with published phylogenetic relationship for annelids, which *N. affinis* was grouped with *Pista cristata* (Terebellida; Terebellidae). This mitogenome resource will be useful for future phylogenetic studies of families belonging to Sedentaria.

The phylum Annelida has two major phylogenetic groups, Pleistoannelida, and six early-branching lineages such as Amphinomida, Chaetopteridae, Lobatocerebrum, Magelonidae, Oweniidae, and Sipuncula (Weigert and Bleidorn 2016), although the phylum is still complex and detailed phylogeny remains debated due to notably derived morphologies, remarkable ecological diversity, and the lack of genomic information (Struck et al. 2011; Weigert et al. 2014; Weigert and Bleidorn 2016; Struck 2017). Two main monophyletic annelid backbones, Sedentaria and Errantia have well-established in Pleistoannelida (Kvist and Siddall 2013; Weigert et al. 2014; Weigert and Bleidorn 2016; Struck 2017), but detailed phylogenetic relationship on families of Sedentaria remained controversial. Members in the sedentarian family Terebellidae are known as spaghetti worms and are ubiquitous tubicolous benthic annelids with morphological and ecological diversity (Stiller et al. 2020). Recently, the monophyly of major taxa including Terebellidae was restored based on transcriptome information and morphological characters (Stiller et al. 2020), whereas there are only two complete mitogenome pieces of information belonging to the family Terebellidae available: *Pista cristata* and *Thelepus plagiostoma* (Nam et al. 2021).

The genus *Neoamphitrite* Hessle 1917, distinguished from the genus *Amphitrite* Müller, 1771 (Hessle 1917) by the morphometric characteristics of branchiae and nephridia (Hessle 1917; Hutchings and Glasby 1988), inhabits soft sediments from intertidal to the shallow offshore reef with a wide range of distribution. In this study, a specimen of *N. affinis* was sampled from the Beaufort Sea (69°52′N, 139°03′W) in 2017 using a remotely operated underwater vehicle (ROV) belonging to the Monterey Bay Aquarium Research Institute (MBARI). All animal handling and experimental procedures were approved by the Animal Welfare Ethical Committee and the Animal Experimental Ethics Committee of the Incheon National University (Incheon, South Korea). The specimen and extracted DNA sample were deposited in the Research Institute of Basic Sciences of Incheon National University (Species ID: Annelid-06; Specimen ID: KOPRI-Benthos-06; https://www.inu.ac.kr/user/indexMain.do?siteId=ribs; Dr. Sang-Eun Nam; se_nam2@inu.ac.kr). Total genomic DNA was isolated from the muscle tissue of a single individual using a DNeasy Blood and Tissue kit (Qiagen, Hilden, Germany) according to the manufacturer’s instructions. Next-generation sequencing was performed to obtain a circular mitogenome with the HiSeq platform (150 bp; HiSeq X ten; Illumina, San Diego, CA, USA) using previously described protocols (Nam et al. 2021). After the quality check, 26,605,410 filtered reads (3,999,082,033 bp) were obtained from 33,789,776 raw reads (5,102,256,176 bp). Thereafter, *de novo* assembly was conducted with various k-mers using SPAdes (Bankevich et al. 2012) to obtain a circular contig of the *N. affinis* mitogenome. The resulting contig consensus sequence was annotated using MITOS2 (Bernt et al. 2013) and tRNAscan-SE 2.0 (Lowe and Eddy 1997). BLAST searches (http://blast.ncbi.nlm.nih.gov) and multiple alignments with terebellids’ mitogenomes confirmed the identity of these genes.

The complete mitogenome of *N. affinis* is 15,823 bp in length (GenBank accession no. MZ326700) with the typical composition, consisting of 13 PCGs, 23 tRNAs, 2 rRNAs, and a large intergenic region presumed to be the control region. However, in addition to the 22 tRNAs, we recover an additional methionine tRNA (*trnM*), which is located next to the first *trnM* gene in tandem. This unique addition of the *trnM* gene in tandem was previously observed in the mitogenomes of *Pista cristata* (Terebellidae) and *Terebellides stroemi* (Trichobranchidae) (Zhong et al. 2008), whereas this phenomenon was not conserved in the family Terebellidae, as *Thelepus plagiostoma* mitogenome contained single *trnM* gene (Nam et al. 2021). Duplication of the *trnM* has also been detected in the mitogenomes of other polychaetes such as *Eurythoe complanata* (Amphinomidae) and *Typosyllis antoni* (Syllidae) (Weigert et al. 2016). The nucleotide composition of *N. affinis* mitogenome was highly biased toward A + T nucleotides (70.3%), with percentages of A, T, C, and G being 33.4%, 37.0%, 18.2%, and 11.5%, respectively. The *COI* sequence showed the highest similarity (99.9%) to the *COI* sequence of *N. affinis* previously registered in GenBank (657 bp; GenBank accession no. MT167006). We constructed the phylogenetic topology of 26 members belonging to Sedentaria and Errantia using the concatenated set of 13 PCG sequences, with two species as an outgroup (Figure 1). *Neoamphitrite affinis* is closely related to *P. cristata* (Terebellidae) within Terebellidae.

**Figure 1.**
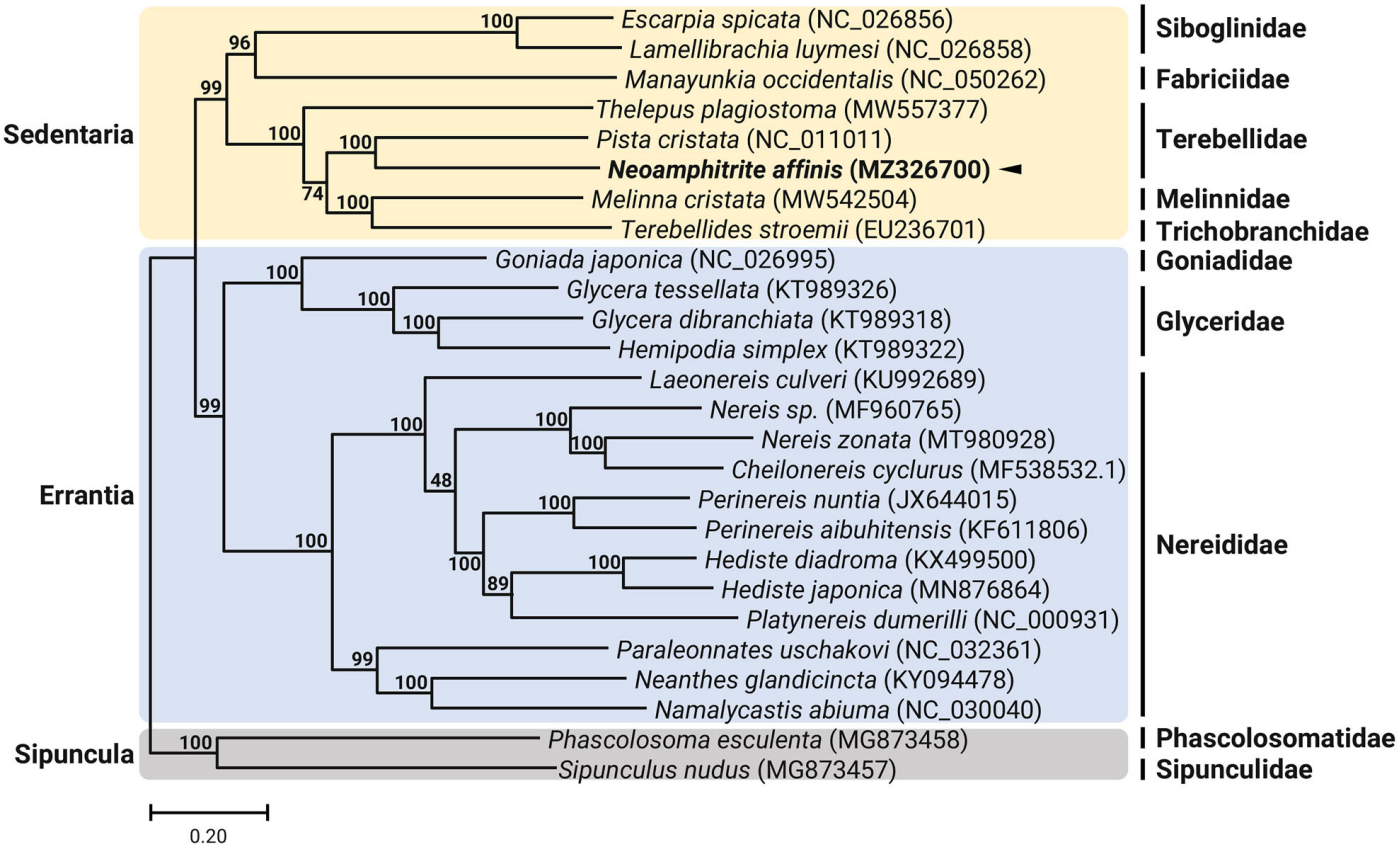
Maximum-likelihood (ML) phylogeny of 8 published mitogenomes from Sedentaria including *T. plagiostoma* and 16 registered mitogenomes of Errantia species, and two Sipuncula species as an outgroup based on the concatenated nucleotide sequences of protein-coding genes (PCGs). The phylogenetic analysis was performed using the maximum-likelihood method, GTR + G+I model with a bootstrap of 1000 replicates. Numbers on the branches indicate ML bootstrap percentages. DDBJ/EMBL/Genbank accession numbers for published sequences are incorporated. The black triangle means the polychaete analyzed in this study.

## Data Availability

BioProject, BioSample, and SRA accession numbers are https://www.ncbi.nlm.ni h.gov/bioproject/PRJNA739841, https://www.ncbi.nlm.nih.gov/biosample/SAMN19803891, and https://www.ncbi.nlm.nih.gov/sra/?term=SRR15041508, respectively. The data that support the findings of this study are openly available in the National Center for Biotechnology Information (NCBI) at https://www.ncbi.nlm.nih.gov, with an accession number MZ326700.
